# Case of Mortification and Separation of the Body of the Uterus; Also an Account of a Monstrous Birth

**Published:** 1807-10

**Authors:** J. Elmer

**Affiliations:** Philadelphia


### *Case of Mortification and Separation of the Body of the Uterus; also an Account of a monstrous Birth. By* Dr. J. Elmer.

A MIDDLE aged woman, the mother of several children, of a slender delicate habit of body, having been exposed to many hardships by reason of her indigent circumstances, was, after hard exercise, taken with a partial *procidentia uteri.* For want of proper assistance, an inflammation and sphacelation of the *fundus uteri* succeeded, and after some days, about the size of a Spanish dollar, of that part of the uterus which was prolapsed without the vagina, separated parated; upon which the inflammation subsided, and the woman soon became well without any help.

About two years afterwards, being obliged to travel several hundred miles in a horse-cart, the fatigue of so long a journey, with the violent jolting of the cart, brought on a complete *procidentia uteri;* the whole uterus falling without the vagina, which was followed with a violent inflammation and swelling of the same, upon which I was sent for. When I visited the patient I found her labouring under a burning fever, sickness at stomach, weakness and great pain in the small of the back, and the prolapsed uterus swelled to the size of a large child's head, as black as a hat, of a cadaverous smell, and with every other mark of an incipient mortification; the poor woman being extremely dejected and despairing of any relief. As her pulse was very small and feeble, and the sphacelation so far advanced, I judged it not advisable to bleed, but ordered the parts to be continually fomented with a strong decoction of bitter herbs, and gave her frequent large doses of a nitrous julep, with a little lavender comp. in it. As she lived at a great distance from me, nothing more was done for her. About three days after I saw her, a separation of the whole body of the uterus, which was now growing putrid, began to take place, and in two or three days more, entirely sloughed off from the sound vagina; upon which the pain and fever abated; the patient recovered her health and strength; and the last time I heard from her, which was several months after this affair happened, she was in perfect health.

---

The following account of an imperfect fœtus may perhaps be thought worthy of notice:

A married woman, in the seventh month of pregnancy, was seized with labour pains that brought on an abortion. She was first delivered of a perfect child, which lived several hours. This being come away, the midwife perceived another behind, of which, with much difficulty, she was also delivered; but instead of a perfect well-formed fœtus, it had the following appearance; the body was completely formed, and about the size of the other child's, but it was wholly destitute of a head, and had but one arm. The body appeared as if the head had been cut square off close to the shoulders; and in the place where the neck shonld be, there was a small spot about the bigness of an English crown, raw and bloody, having a fibrous appearance as though it had adhered to something.

 The

The side on which the arm was wanting was perfectly smooth without any vestige of an arm. The other arm was well-formed as far as the hand, but it had no fingers, only two little fleshy excrescences, about half an inch long, growing in the place of fingers. Its legs were well shaped, but its heels grew where the instep ought to be, and it was destitute of toes, having only two fleshy excrescences on each foot, (similar to those on the hand) in place of toes. It had no umbilical cord nor placenta annexed to it. In the place where the umbilicus ought to be, or rather a little higher, there was a small fleshy production about two inches long, which being cut off, appeared white and bloodless, having no marks of blood vessels in it. The flesh of its body appeared bloated, and felt harder than usual, and its joints were so stiff as to be bent with difficulty.

I had no opportunity of examining it thoroughly, but am of opinion it adhered to some part of the placenta of the other fœtus, in the place where it appeared raw and lacerated, and that it received its nourishment *in utero*, by the small blood vessels in that part inosculating with those of the placenta.

Such imperfect productions as this should teach us not to draw too hasty conclusions from preternatural appearances. The dispute whether the fœtus *in utero* receives its nourishment by the mouth or umbilical vessels, or both, has long subsisted, and many arguments have been deduced on both sides the question from the mal-formation of the chylopoietic organs of fœtuses. In the present instance, it is very evident that it received its nourishment neither by the mouth nor umbilicus, for both of them were wanting. It must therefore have received it either in the manner some of the ancients suppose the fœtus to be nourished, viz. by absorbing the nutritious particles through the pores of the body in the same manner as a sponge imbibes water, or else as above conjectured. The latter will perhaps appear the most probable.

J. ELMER.

*Philadelphia, September 14, 1773.*

*A Case*

